# Prediction of CTCF loop anchor based on machine learning

**DOI:** 10.3389/fgene.2023.1181956

**Published:** 2023-04-03

**Authors:** Xiao Zhang, Wen Zhu, Huimin Sun, Yijie Ding, Li Liu

**Affiliations:** ^1^ School of Mathematics and Statistics, Hainan Normal University, Haikou, China; ^2^ Yangtze Delta Region Institute (Quzhou), University of Electronic Science and Technology of China, Quzhou, China; ^3^ Key Laboratory of Computational Science and Application of Hainan Province, Haikou, China; ^4^ School of Physical Science and Technology, Inner Mongolia University, Hohhot, China

**Keywords:** CTCF, Chromatin Loop, Machine Learning, DNA sequence, 3D Genome

## Abstract

**Introduction:** Various activities in biological cells are affected by three-dimensional genome structure. The insulators play an important role in the organization of higher-order structure. CTCF is a representative of mammalian insulators, which can produce barriers to prevent the continuous extrusion of chromatin loop. As a multifunctional protein, CTCF has tens of thousands of binding sites in the genome, but only a portion of them can be used as anchors of chromatin loops. It is still unclear how cells select the anchor in the process of chromatin looping.

**Methods:** In this paper, a comparative analysis is performed to investigate the sequence preference and binding strength of anchor and non-anchor CTCF binding sites. Furthermore, a machine learning model based on the CTCF binding intensity and DNA sequence is proposed to predict which CTCF sites can form chromatin loop anchors.

**Results:** The accuracy of the machine learning model that we constructed for predicting the anchor of the chromatin loop mediated by CTCF reached 0.8646. And we find that the formation of loop anchor is mainly influenced by the CTCF binding strength and binding pattern (which can be interpreted as the binding of different zinc fingers).

**Discussion:** In conclusion, our results suggest that The CTCF core motif and it’s flanking sequence may be responsible for the binding specificity. This work contributes to understanding the mechanism of loop anchor selection and provides a reference for the prediction of CTCF-mediated chromatin loops.

## 1 Introduction

High-order chromatin structure influences a variety of biological processes in the nucleus, including gene transcription, gene regulation and DNA replication. The structure of interphase chromatin has been extensively researched with the development of various chromatin conformation capture techniques (Fullwood et al., 2009a; Fullwood et al., 2009b; Lieberman-Aiden et al., 2009; Hsieh et al., 2015), unveiling the functional units. For example, extensive researches on chromosome compartments (Dixon et al., 2012), topologically associated domains (TADs) (Rao et al., 2014) and loops (Narendra et al., 2015) have been carried out. Chromatin loops usually form between the locus that separated by hundreds of thousands base pairs. These long-range interactions usually form a local chromatin structure. According to previous studies, the destruction of these loops leads to a significant imbalance in nearby gene expression (Lupiáñez et al., 2015; Hnisz et al., 2016). The binding sites of CTCF (an 11 zinc finger DNA binding protein) frequently occur on the boundaries of loops and topologically associated domains, which highlights the importance of CTCF binding for the loop formation.

In the process of gene expression, the gene regulatory elements work in order. These regulatory elements can be classified as promoters, enhancers, insulators, and other regulatory sequences (West et al., 2002; Kellis et al., 2014). Insulators protect genes in cells from inappropriate regulatory signals from adjacent chromatin environments, and play an important role in cell type-specific gene expression (Liu et al., 2019). CTCF was originally thought to be an active chromatin-labeled insulator. As an evolutionarily conserved zinc finger family transcription factor, CTCF was discovered for the first time in the chicken gene promoter (Bell and Felsenfeld, 2000). CTCF was found to be related to blocking the activity of enhancers in the process of transcription (Liu et al., 2021). Changes in the CTCF protein and its binding sites on insulators are linked to a variety of human diseases. For example, deletion of CTCF in the domain may result in an interaction between the enhancer and a glioma oncogene (Katainen et al., 2015); the binding site of CTCF is the main mutation hot spot of the non-coding cancer genome (Ohlsson et al., 2001); zinc finger mutation or abnormal target selective methylation destroy the spectrum of target specificity and is related to cancer (Phillips and Corces, 2009).

CTCF was later found to play an important role in chromatin organization. Paired CTCFs binding act as loop anchors to limit the interaction between remote regulatory elements (de Wit et al., 2015; Rowley and Corces, 2018). As a result, how to distinguish the interacting CTCF pair and the non-interacting CTCF pair is a critical issue. Many experiments have revealed that the interaction between CTCF and cohesin is crucial for loop formation (Wutz et al., 2017). This interaction establishes a dynamic chromatin loop between remote CTCF binding sites to drive the formation of TADs. The chromatin loops may form through the process of loop extrusion (Alipour and Marko, 2012; Barbieri et al., 2012; Fudenberg et al., 2016; Haarhuis et al., 2017; Rao et al., 2017; Davidson et al., 2019; Kim et al., 2019). Cohesin can pass through and extrude DNA to form chromatin loops until it is blocked by CTCF. In addition, the formation of the loop can also be realized through other mechanisms (Brackley et al., 2013; Bianco et al., 2018; Conte et al., 2020). Although the formation mechanism of the loop has been deeply studied, the ability of the model based on polymer physics to predict a single CTCF loop has not been systematically evaluated (Di Pierro et al., 2016; Kai et al., 2018). The machine learning model named Lollipop uses 77 features of the genome and epigenome to predict the interaction of CTCF pairs (Lv et al., 2021). Deep-loop uses only DNA sequences to predict CTCF-mediated chromatin loops (Xi and Beer, 2021). The loop extrusion and competition model can predict the specificity of CTCF interaction through four characteristics. These four characteristics are chromatin loop competition, CTCF binding site distance, CTCF motif and CTCF binding intensity. The aforementioned experiments aim to predict the loops formed between pairs of CTCF binding sites and require the CTCF ChIP-seq data as input. In mammalian cells, there are approximately 50,000 CTCF binding sites, corresponding to more than one million possible CTCF pairs separated by less than 1 Mb. However, Hi-C or ChIA-PET measurements revealed that only approximately 2%–5% of CTCF pairs are directly interacting. This increases the difficulty of *de novo* prediction task. We notice that only a portion of CTCF binding sites are used as loop anchors. Can we first distinguish the loop anchor and non-anchor to reduce the search space for loop identification? Motivated by this idea, we intend to determine if a single CTCF binding site may serve as the anchor of loop by using sequence and binding intensity features. We find the binding intensity of CTCF, the core motif and the flanking sequence of the motif all have an important influence. Previous models ignore the flanking sequence features of the CTCF motif. In this paper, we developed support vector machine (SVM) (Guo et al., 2008; Zhang and Liu, 2017), convolutional neural network (CNN) (Li and Liu, 2020; Cui et al., 2021), random forest (RF) (Xu et al., 2019; Dao et al., 2022), linear discriminant analysis (LDA), Naive Bayes (NB), logistic regression (LR) (Yang et al., 2021) and stochastic gradient descent (SGD) model to predict the potential of CTCF binding to form chromatin loop anchors. We considered the binding intensity of CTCF, the sequence characteristics of the CTCF core motif and the flanking sequence as input features. These features performed well in almost all the models, indicating that they are important for the formation of loop anchors.

## 2 Materials and methods

### 2.1 Data source

We download the public ChIP-seq data of CTCF from the ENCODE database (Ecker et al., 2012). The detection method is ChIP-seq, the target set is transcription factors, the biological sample term is GM12878, the reference genome is hg19, and the file type is bed narrowPeak. We also downloaded ChIA-PET data of CTCF from ENCODE. The detection method is ChIA-PET, the target set is transcription factors, the biological sample term is GM12878, the reference genome is hg19, and the file type is fastq.

### 2.2 Data processing

The positive and negative set was constructed as follows: First, ChIA-PET data of CTCF were preprocessed by using ChIA-PET2 (Li et al., 2017). ChIA-PET2 can significantly improve the sensitivity and reproducibility of detecting chromatin loops while maintaining the same false discovery rate. We can calculate the false discovery rate of each ChIA-PET data by ChIA-PET2. The false discovery rate refers to the expected value of the proportion of the number of falsely rejected true assumptions compared to the number of rejected original assumptions. The false discovery rate offers several advantages, including flexible adjustment of its value, clear meaning, and its ability to be used as an evaluation metric for screened different variables. The ChIA-PET data of CTCF will give a pair of DNA anchors that can form chromatin loops. Here, the data with FDR < 0.05 are considered as the CTCF-mediated chromatin loops. In addition, we focus on whether single CTCF site can form loop anchor. ChIA-PET data consists of a combination of two anchors, which can result in a single anchor corresponding to multiple other anchors. Therefore, when extracting location data of anchors, repeated anchors may be generated. Thus, after removing duplicate anchors in the data with the cutoff of FDR < 0.05, we obtained the location data of all CTCF loop anchors in the GM12878 cell line. Secondly, the ChIP-seq data of CTCF were used to obtain the location of the core motif with a length of 19 bp at the corresponding data by storm (Schones et al., 2007). Storm scans the input sequences and find the fragment with the highest motif score. Importantly, it also provides the information about whether sequence fragment occurs on the sense chain or antisense chain. This data will later be crucial for extracting sequences of CTCFs in the same binding direction. Finally, The motif location data were compared with the CTCF loop anchors. If there is an overlap between them, it is considered that the binding site of the CTCF can form loop. Then, the position data of these motifs are taken as the positive set and add the label “1”. The position data of non-overlapping motifs are taken as the negative set that cannot form a loop, and the label “0”is added. In the GM12878 cell line, the number of samples that cannot form loops is 26,765, the number of samples that can form loops is 22,191, and the total number of samples is 48,956.

The context sequence of CTCF, in addition to its core motif, is crucial for controlling gene expression. Huang et al. (2021) used the SOX2 gene reporting system of mouse embryonic stem cells to study how the context sequence of the CTCF binding site regulates insulator function. They discovered the following: 1) The 10–20 bp sequence upstream of the core motif of CTCF rather than the core motif itself determines whether CTCF can perform the insulator function 2) The insulating effect depends on the number of CTCF tandem binding sites. These findings provide new insights into the classification of CTCF binding sites. The binding and dissociation of CTCF on the genome is a dynamic process. The residence time of CTCF is determined by the binding stability. CTCF has 11 zinc finger structures. The zinc finger ZF3-ZF7 binds with the core motif, and ZF9-ZF11 binds 10–20 bp upstream of the core motif. The existence of ZF8 as a linker also plays an important role in promoting the overall binding stability (Soochit et al., 2021). In the above experiment, when each flanking sequence of the motif gradually decreases from 60 bp to 20 bp, the insulation effect does not decrease significantly, and the strong insulation effect of CTCF always exists. However, when the flanking sequence of the core motif gradually decreases to 10 bp, the strong insulation effect of CTCF is significantly reduced. This demonstrates that the flanking sequences 10–20 bp from core motif has a significant effect on the insulation effect. Furthermore, the bases upstream and downstream of the motif will have a great impact on the function of CTCF. Therefore, we added 20 bp upstream and downstream to the CTCF motif and obtained the location data of the 59 bp sequence. Because the binding of CTCF is directional, the sequence direction should be taken into account when extracting sequences. One-hot encoding is used to make the 59 bp sequence fragment into a matrix consisting of 0 and 1, where base A corresponds to (1,0,0,0), base T corresponds to (0,1,0,0), and bases C and G correspond to (0,0,1,0) and (0,0,0,1), respectively. Then, a 48,956 × 236 one-hot matrix is obtained.

The binding intensity of CTCF can affect the movement of cohesin, thereby affecting the formation of the loops. The narrowPeak ChIP-seq data gives the CTCF binding intensity at the corresponding position. There is a large variation among the CTCF binding intensity values. It will greatly affect the training of the model. Due to the unique characteristics of each assessment index, a multi-index evaluation system typically has different dimensions and orders of magnitude. If the original indicator values are used for analysis when there is significant variation between the indicators, the importance of the indicators with higher values will be accentuated, while the significance of the indicators with lower values will be substantially diminished. Therefore, data normalization is required for the CTCF binding intensity to reduce the impact of the large variation in the training model. Here, we took the logarithm base two of the CTCF binding intensity value to narrow the gap between the value of CTCF binding intensity with the sequence data. Finally, the normalized value of the CTCF binding intensity and one-hot matrix were merged to construct the feature matrix (Supplementary Material).

### 2.3 Summary of the machine learning model

The basic motivation of the support vector machine (SVM) is to find a decision hyper plane to maximize the interval between the two types of data, construct an objective function according to the maximum interval, and then transform it into its dual problem for solution. For non-linear problems, first use a transformation z = φ (x) to map x to a new feature space z, then transform it into the dual problem of support vector machine, and we use radial basis functions as kernel functions.

The random forest (RF) algorithm is an ensemble algorithm composed of multiple decision tree classifiers, with each subclassifier being a CART classification regression tree. Therefore, random forest can perform both classification and regression. The risk of overfitting can be reduced by averaging the decision trees.

Convolutional Neural Network (CNN) is a specialized type of artificial neural network commonly used in deep learning for analyzing visual imagery. It is designed to automatically and adaptively learn spatial hierarchies of features from input images or other two-dimensional data, such as audio spectrograms. CNNs are composed of multiple convolutional layers that apply mathematical operations called convolution to the input data, followed by pooling layers that reduce the dimensionality of the output from the convolutional layers. The output of the pooling layers is then fed into fully connected layers, which perform the final classification or regression of the input data.

We also use other machine learning models to train on the same dataset, including the linear discriminant analysis (LDA), Naive Bayes (NB): The Naive Bayes method is a classification technique that is based on Bayes’ theorem and the assumption of independently occurring features, logistic regression (LR): logistic regression is a generalized linear regression that utilizes logistic functions, and stochastic gradient descent (SGD): stochastic gradient descent is an iterative optimization algorithm used to update a model’s parameters based on the steepest descent direction of the loss function.

### 2.4 Performance assessment

We have employed several machine learning models to train on the same dataset, including linear discriminant analysis (LDA), support vector machines, Random Forest (RF)), logistic regression (LR), stochastic gradient descent (SGD), Naive Bayes (NB) models, convolutional neural networks (CNN), support vector machine models (SVM). We then compared the results of each model.

To evaluate the prediction performance of the model, Accuracy (Acc), Precision (Pre), F1-score (F1), Area Under ROC Curve (AUROC), Area Under PRC Curve (AUPRC), Specificity (Sp), Sensitivity (Sn) and Matthews correlation coefficient (MCC) are used as evaluation indicators (Zhang Q. et al., 2022; Zhang Z. Y. et al., 2022; Han et al., 2022; Yang et al., 2022). TP (True Positive): successful prediction of positive samples as positive. FP (False-Positive): incorrectly predicts negative samples to be positive. TN and FN correspond to the value of the negative set.

The ratio of correctly classified positive samples in the total number of positive samples:

$$S_{n}=\frac{TP}{TP+FN}$$ (1)

The ratio of correctly classified negative samples in the total number of negative samples:

$$S_{p}=\frac{TN}{TN+FP}$$ (2)

MCC, which has a value range of [- 1,1], is simply a correlation coefficient that describes the relationship between actual classification and prediction classification. A score of 1 denotes the subject’s perfect prediction, a value of 0 denotes that the prediction result is less accurate than a random prediction, and a value of −1 denotes that there is no consistency between the predicted classification and the actual classification:

$$MCC=\frac{TP\times TN-FP\times FN}{\sqrt{\left(TP+FP\right)\left(TP+FN\right)\left(TN+FP\right)\left(TN+FN\right)}}$$ (3)

The ratio of the sample size of correctly classified positive samples to the total number of samples predicted by the model as positive samples:

$$\mathrm{Pr}ecision=\frac{TP}{TP+FP}$$ (4)

The F1 score is the harmonic average of precision and recall:

$$F1=2\times \frac{\mathrm{Pr}ecision\times Recall}{\mathrm{Pr}ecision+Recall}$$ (5)

## 3 Results

### 3.1 Overview of CTCF loop anchor prediction

**FIGURE 1:**
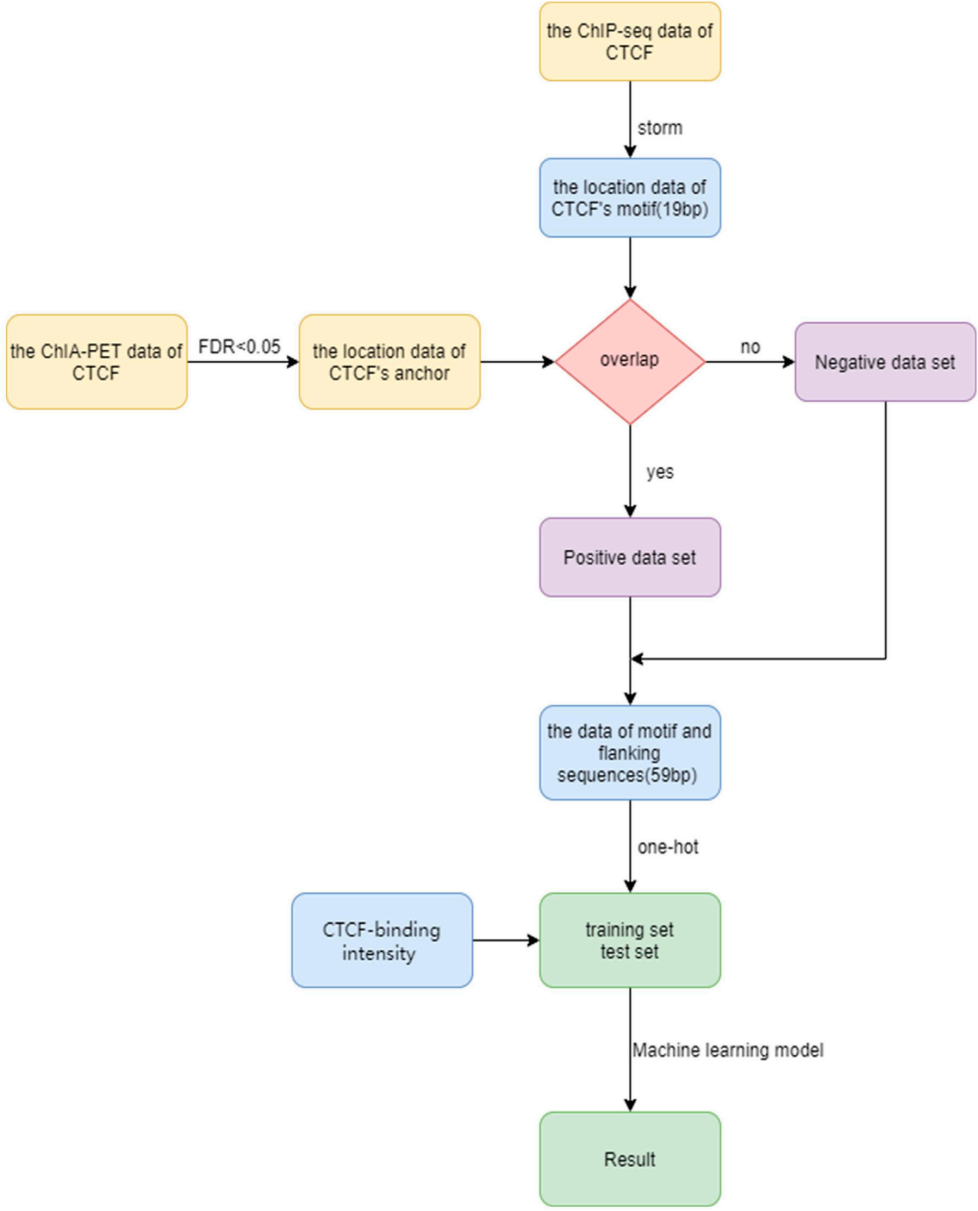
Flow chart for CTCF loop anchor prediction.

In order to predict the CTCF loop anchor, we propose a computational framework (Figure 1). The framework includes dataset construction, feature extraction, and machine learning algorithm selection. We first establish the precise location of the CTCF binding sites based on ChIP-seq data and motif scanning. The positive and negative sets are then generated based on ChIA-PET data. The feature matrix is constructed by extracting sequence of the core motif, flanking sequence, and CTCF binding strength. The machine learning methods are implemented on the feature matrix to distinguish the loop anchor and non-anchor. More details of the framework are discussed in the Materials and methods section.

### 3.2 Comparison of prediction performance

**FIGURE 2:**
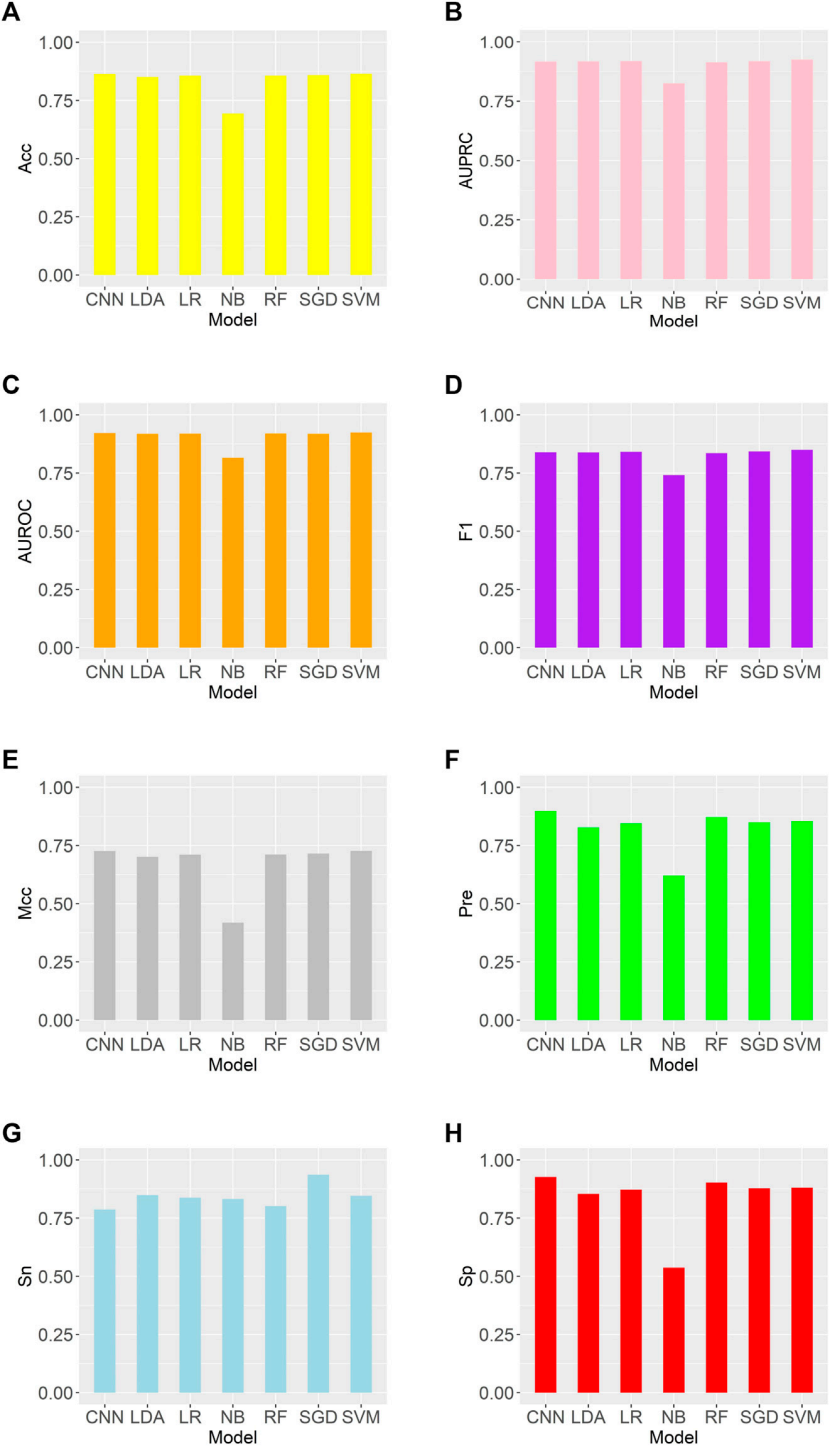
Comparison of evaluation criteria between support vector machine and other machine learning models **(A–H)** are as follows: Acc, AUROC, AUPRC, F1, MCC, Pre, Sn, Sp.

**FIGURE 3:**
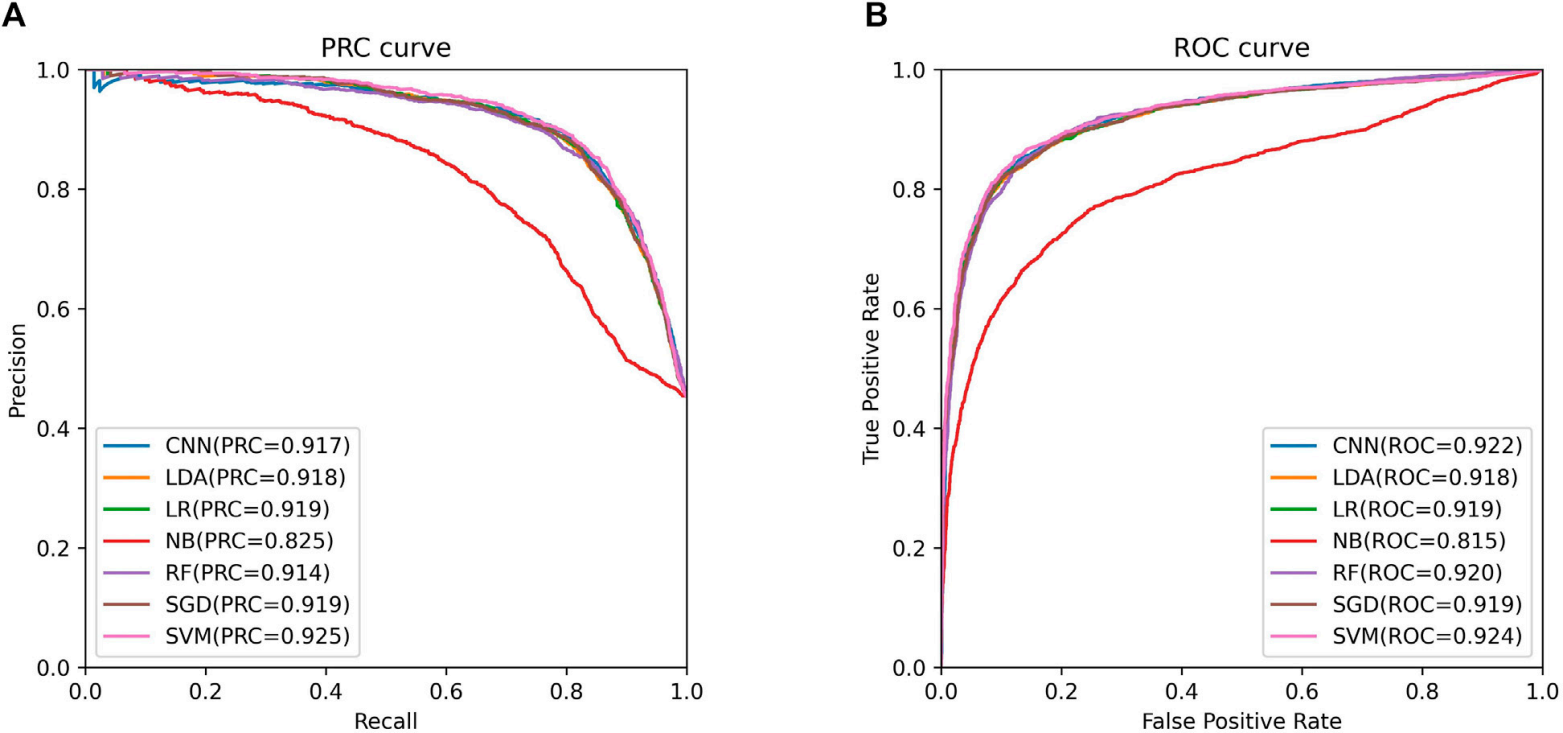
ROC and PRC curves of support vector machine model. **(A)**. The PRC curve of support vector machine model. **(B)**. The ROC curve of support vector machine model.

We trained these machine learning models by using tenfold cross-validation, and then tested the prediction performance on a separated independent testing set. The training dataset is randomly divided into K subgroups of the same size for the K-fold cross validation test. The remaining K-1 folds are utilized as the training dataset for the machine learning model, while one fold is used as the validation dataset. Each fold serves as the validation dataset once this procedure has been repeated K times. One-10th of the dataset is used as an independent testing set, and the rest is considered as a training set. The training set was used to perform ten-fold cross-validation and train the model, and then the model’s performance is verified on the test set. We compared the predictive performance of seven machine learning models on independent test sets by evaluating Sn, Sp, Pre, Acc, MCC, F1, AUROC, and AUPRC (Figure 2). Except for the Naive Bayes model, the accuracy rates of the other models are greater than 0.85, with the support vector machine model having the highest accuracy rate of 0.8646. As shown in Figure 3, the AUROC and AUPRC values of the other models (in addition to the naive bayes model) are around 0.92, and they perform well in terms of the remaining F1 score, precision, and other evaluation criteria. This demonstrates that the three types of features we selected have a good predictive effect across a variety of different machine learning models. The good performance of the selected features indicates they have an important influence on the process of CTCF binding to form chromatin loops.

### 3.3 Importance of features

We found no significant difference in the prediction results obtained from the SVM, SGD, RF, LR, LDA, and CNN models. So we selected the SVM model, which had a slight advantage in results, and used different features and feature combinations for prediction. To assess the contribution of various features or feature combinations to the prediction, we used the core motif, flanking sequence, and their combination with CTCF binding intensity as the features to perform prediction (Table 1). We have discovered that CTCF binding intensity alone has good predictive performance, indicating its important role in the loop formation process. Although the flanking sequence has slightly lower prediction accuracy compared to the core motif, the combinations of the flanking sequence and CTCF binding intensity yields a marginally better outcome than the combination of core motif and binding intensity. This demonstrates that the CTCF binding intensity and core motif features are somewhat redundant. Adding the flanking sequence feature to the model can increase prediction accuracy.

**TABLE 1:** Prediction performance of different feature and combinations of features by SVM model.

	Acc	AUROC	AUPRC
Core motif	0.6945	0.7491	0.7184
Flanking sequence	0.6467	0.6855	0.6422
CTCF binding intensity	0.8375	0.8954	0.8916
Core motif and CTCF binding intensity	0.8458	0.9118	0.9061
Flanking sequence and CTCF binding intensity	0.8599	0.9225	0.9226

**FIGURE 4:**
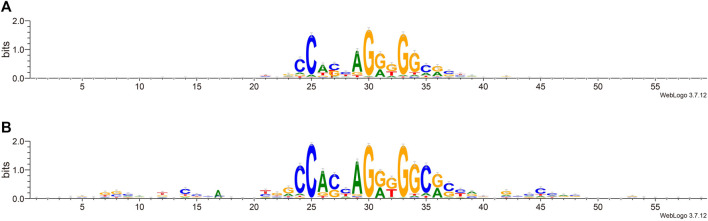
Sequence alignment of positive and negative sets. **(A)**. The negative set. **(B)**. The positive set.

We next try to find the key sites that play an important role in the classification. We calculated the information content of each site by using weblogo (Crooks et al., 2004). From Figure 4, we can see that in the flanking sequence of the core motif, there are obvious differences between the positive set and negative set of sequence data, which is consistent with previous views (Huang et al., 2021; Soochit et al., 2021). The flanking sequence also plays an important role in the process of CTCF binding, and it will affect whether CTCF can be used as the loop anchor. Comparing the information content shows that positive sets prefer certain flanking sequence sites: 7–9, 12, 14, and 42–48. This further reveals that the flanking sequence feature can effectively distinguish chromatin loop anchor.

**FIGURE 5:**
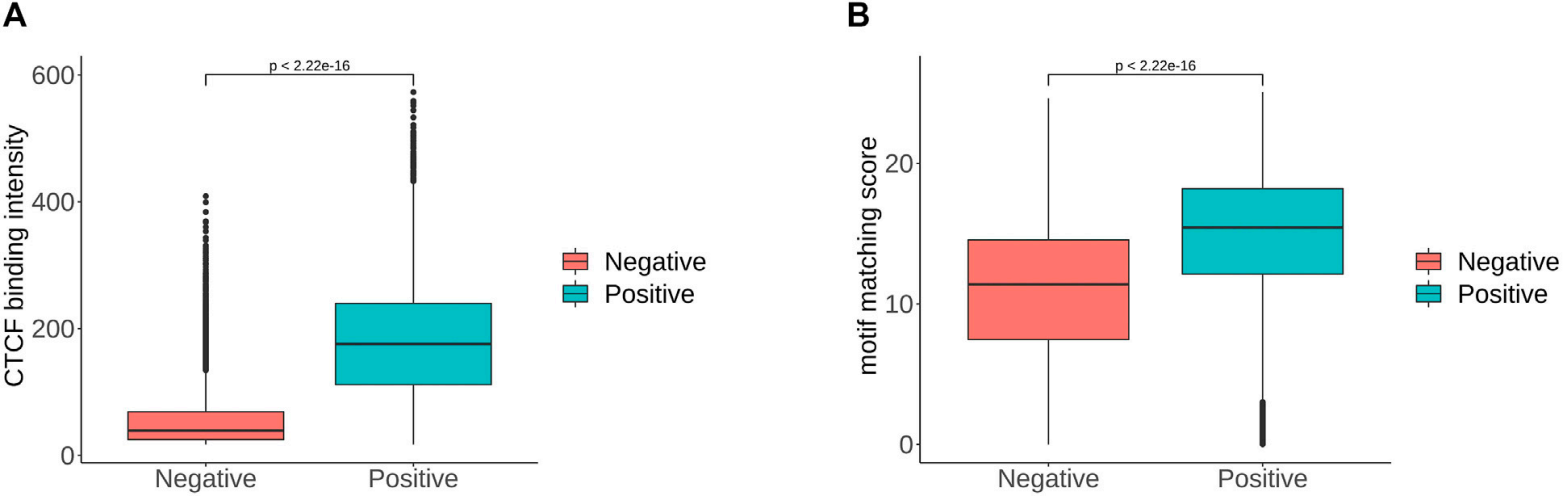
**(A)**. CTCF binding intensity of positive and negative sets. **(B)**. CTCF motif matching score of positive and negative sets, where the *p*-value is given by t-test.

In order to determine the importance of each feature more precisely, we performed feature selection, sometimes referred to as feature subset selection (FSS). It alludes to the process of choosing N characteristics from the already-existing M features to optimize the system’s particular indicators. To decrease the dimension of the dataset and enhance the efficiency of the learning algorithm, it is necessary to choose some of the most useful characteristics from the original features. The generating process, evaluation function, stop criteria, and verification procedure are the four main components of the feature selection process. We selected the top 20 features in order of importance, and the most important feature was the value of the CTCF binding intensity corresponding to the sequence. We ranked the significance of the features and find that the flanking sequence sites 12, 45, 46 and 47 significantly contribute to classification. The sites 45 to 47 correspond to CTCF zinc fingers 1 to 3. Based on the analysis combined with Figure 4, there were significant sequence differences observed between the positive and negative sets at the flanking sites 12–17. These sites corresponded to the binding region of zinc finger 8–11 of CTCF. There are 11 zinc fingers in CTCF, and not all of them bind to DNA at the same time. According to the research of Soochit et al. (2021), the removal of zinc finger 8 results in a decrease of chromatin residence time. Our result also suggests that the flanking sequence may influence the residence time of CTCF on DNA. Additionally, we compared the CTCF binding intensity and motif matching score for positive and negative sets The CTCF binding intensity of the positive set was mostly greater than that of the negative set (Figure 5A). The same is true for the CTCF motif matching score calculated by storm (Figure 5B). The motif matching score is a method used to measure the similarity between a query motif and a target motif. The positive set had a higher matching score, suggesting that the binding of CTCF would be more stable. The results support that the stronger the binding intensity, the more it can prevent the movement of cohesin and thus form loops. Therefore, the CTCF binding intensity is indeed an important feature to reflect whether CTCF can be used as the anchor for forming loops.

From the research results, it can be concluded that the most important factor affecting the formation of loop anchor is the CTCF binding intensity. The sequence preference of flanking sequence and the motif matching score are consistent with the different distributions of CTCF binding strength in the positive and negative sets. A more suitable sequence context is favorable to the stable binding of CTCF and makes it simpler to prevent the sliding of cohesin and thus form a loop anchor. The statistical analysis of these three characteristics revealed that CTCF binding strength, core motif, and flanking sequence are the most important factors in predicting loop anchor.

## 4 Discussion

As an important transcriptional regulation mechanism in organisms, the process of chromatin looping has been widely studied. Previous studies have shown that this process can be interpreted by the loop extrusion model (Xi and Beer, 2021). The details of mechanism are gradually dissected. For instance, the recent study demonstrates that the flanking sequence of CTCF motif have a major impact on the TAD border formation (Huang et al., 2021). Motivated by the experimental results and our statistical analysis, we try to answer which CTCF binding sites may form loop anchors. Our analyses indicate that the CTCF binding intensity, the core motif sequence and the flanking sequence have a certain difference between CTCF loop anchors and non-anchors. Using these features, we employed machine learning models to predict CTCF loop anchors. We conducted ten-fold cross-validation and independent testing, both of which demonstrated the ability of these characteristics to produce accurate prediction results, The statistical analysis showed a significant difference in CTCF binding strength between the positive and negative sets, as well as in the motif matching score. These results indicate that CTCF binding strength can be used as a classification feature. Moreover, this difference may be influenced by the motif and flanker sequences, highlighting their importance as features for predicting CTCF loop anchors. Specifically, based on feature importance ranking, we have identified the flanking sequence sites 12 and 45 to 47, which are likely bound by CTCF ZF8 and ZF1-3, make a significant contribution. This is consistent with other study (Soochit et al., 2021) that the upstream and downstream motifs determine the stability of CTCF binding to DNA. In conclusion, our results suggest that a better sequence context is favorable to the stable binding of CTCF and makes it easier to block loop extrusion by cohesin. Our study provides new insights into the functional classification of CTCF and might even be helpful for the prediction of CTCF-mediated chromatin loops.

## Data Availability

The original contributions presented in the study are included in the article/Supplementary Material, further inquiries can be directed to the corresponding author.
